# Electrochemiluminescence Biosensors Using Screen-Printed Electrodes

**DOI:** 10.3390/bios10090118

**Published:** 2020-09-09

**Authors:** Emiliano Martínez-Periñán, Cristina Gutiérrez-Sánchez, Tania García-Mendiola, Encarnación Lorenzo

**Affiliations:** 1 Departamento de Química Analítica y Análisis Instrumental Universidad Autónoma de Madrid, 28049 Madrid, Spain; emiliano.martinez@uam.es (E.M.-P.); cristina.gutierrezs@uam.es (C.G.-S.); tania.garcia@uam.es (T.G.-M.); 2 Institute for Advanced Research in Chemical Sciences (IAdChem) Universidad Autónoma de Madrid, 28049 Madrid, Spain; 3 IMDEA-Nanociencia, Ciudad Universitaria de Cantoblanco, 28049 Madrid, Spain

**Keywords:** screen-printed electrodes, nanomaterials, enzymatic biosensor, immunosensor, DNA sensor

## Abstract

Electrogenerated chemiluminescence (also called electrochemiluminescence (ECL)) has become a great focus of attention in different fields of analysis, mainly as a consequence of the potential remarkably high sensitivity and wide dynamic range. In the particular case of sensing applications, ECL biosensor unites the benefits of the high selectivity of biological recognition elements and the high sensitivity of ECL analysis methods. Hence, it is a powerful analytical device for sensitive detection of different analytes of interest in medical prognosis and diagnosis, food control and environment. These wide range of applications are increased by the introduction of screen-printed electrodes (SPEs). Disposable SPE-based biosensors cover the need to perform in-situ measurements with portable devices quickly and accurately. In this review, we sum up the latest biosensing applications and current progress on ECL bioanalysis combined with disposable SPEs in the field of bio affinity ECL sensors including immunosensors, DNA analysis and catalytic ECL sensors. Furthermore, the integration of nanomaterials with particular physical and chemical properties in the ECL biosensing systems has improved tremendously their sensitivity and overall performance, being one of the most appropriates research fields for the development of highly sensitive ECL biosensor devices.

## 1. Introduction

**Scheme 1:**
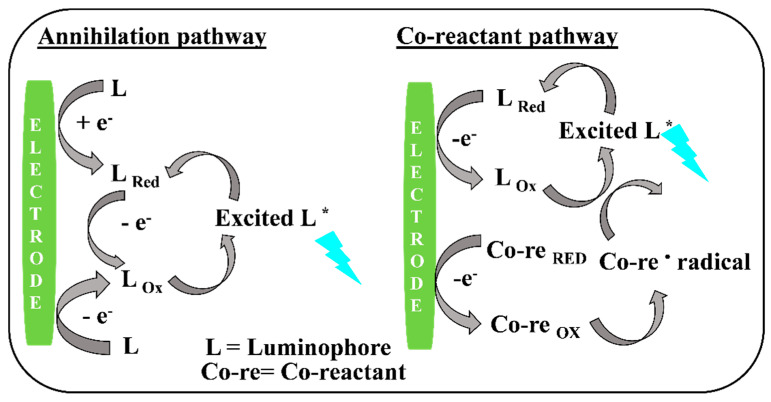
ECL mechanisms: Annihilation and co-reactant pathway.

Electrochemiluminescence (ECL) is a chemiluminescence phenomenon resulting from the electrochemical excitation of a luminescence system (luminophore) that emits light when it returns to its fundamental state [1]. The mechanisms associated with these phenomena are well known and described in the literature, being classified into two main types [2], as can be seen in Scheme 1.

The annihilation pathway: A reduced specie and an oxidized specie (charged radical ions) are simultaneously generated at the electrode surface by applying alternating pulse potentials. These two species react between them generating an excited form, which in the relaxation process to the ground state emits a photon [3].

Co-reactant pathway: A co-reactant is a chemical specie that is reduced or oxidized at the electrode surface, generating a very reactive intermediates that react with the reduced or oxidized luminophore (specie capable of emit light) present in the solution to produce the excited state. Finally, the excited state returns to the ground state to cause chemiluminescence. Employing a co-reactant is especially useful when either radical charged ions are not stable enough for the ECL annihilation reaction, or radical ions cannot both be formed because of the solvent has a narrow potential window. With a co-reactant ECL can be generated by applying a potential in one direction. There are two reaction paths to produce the excited state of the ECL emitter, reductive-oxidation or oxidative-reduction ECL. For instance, oxalate ion (C_2_O_4_^2−^) [4,5] and several amines [6,7,8,9] can be used for oxidative-reduction ECL where an oxidative step produces a strong reductant, whereas peroxidisulfate ion (S_2_O_8_^2−^) [10,11,12] is frequently used for reductive-oxidation ECL.

As described above, ECL reactions require a luminophore. Even though many compounds have been demonstrated to participate in ECL reactions, most of them require aprotic and deoxygenated solution conditions. Therefore, only few compounds and their derivatives are primarily utilized for aqueous-based ECL bioanalytical detection methods. These are luminol (5-amino-2,3-dihydrophthalazine-1,4-dione) and ruthenium (II) chelates [RuL_3_]^2+^. Nowadays, new luminophores such as semiconductor nanomaterials are being widely used with great results. This fact is one of the main reasons ECL sensor and biosensor are having a great and successful advance.

Bioanalytical methods based on [RuL_3_]^2+^ ECL were not developed until the co-reactant pathway was reported between Tris(bipyridine) ruthenium (II) [Ru(bpy)_3_]^2+^ and oxalate [5] in aqueous media and unaffected by the presence of oxygen was reported. [Ru(bpy)_3_]^2+^ is a model luminophore that is largely used nowadays; the discovery of its ECL emission in aqueous media with efficient co-reactants such as tri-n-propylamine (TPrA) [8,13] has led to successfully bioassays for clinical diagnosis. Probably one of the most relevant co-reactant pathways is the “oxidative–reductive” system between aliphatic amines and [RuL_3_]^2+^ as [Ru(bpy)_3_]^2+^. In this mechanism, both [RuL_3_]^2+^ and the co-reactant are oxidized (Equations (1) and (2)). The TPrA radical cation is unstable on the time frame of the experiment and quickly deprotonates, forming a free radical, TPrA* (Equation (3)). This free radical behaves as a reducing agent and transfers an electron to [RuL_3_]^3+^, producing an excited state (Equation (4)).

(1) $${\left[{\mathrm{RuL}}_{3}\right]}^{2+}+1e^{-}\to \;{\left[{\mathrm{RuL}}_{3}\right]}^{3+}$$

(2) $$\mathrm{TPrA}\;\to {\;\mathrm{TPrA}}^{+*}\;+1e^{-}$$

(3) $${\mathrm{TPrA}}^{+*}\;\to {\;\mathrm{TPrA}}^{*}+H^{+}$$

(4) $${\mathrm{TPrA}}^{*}+\;{\left[{\mathrm{RuL}}_{3}\right]}^{3+}\;\to \;{\left[{\mathrm{RuL}}_{3}\right]}^{2+*}+\mathrm{products}$$

**Figure 1:**
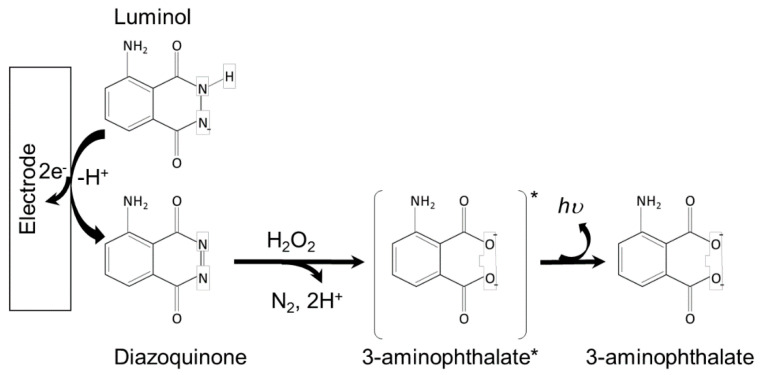
Scheme showing the ECL mechanism of luminol with hydrogen peroxide. Reproduced from reference [14]. Reproduced and adapted with permission of MDPI.

As [Ru(bpy)_3_]^2+^, luminol is one of the most used reagents in ECL applications [1,14] in aqueous alkaline solutions. Luminol is oxidized at the electrode surface and forms a diazaquinone intermediate, which reacts quantitatively with hydrogen peroxide to produce the 3-aminophtalate in an excited state due to O–O bond cleavage in the endoperoxide form. 3-Aminophthalate then emits a characteristic blue light at 425 nm (see Figure 1) [14,15].

Different mechanistic pathways have been suggested depending on the applied electrode potentials. In addition, the ECL intensity correlates directly with the amount of hydrogen peroxide. The luminol ECL method can be used to determine either luminol or species labeled with luminol or peroxides. Since hydrogen peroxide is an analyte of interest in various biological applications, this luminophore can measure reactive oxygen species. In addition, as we describe below, the enzymatic activity of oxidase-type enzymes, which generate hydrogen peroxide in the presence of their substrates, can be monitored using luminol.

In electrochemiluminescence assays, the energy of the excitation source (electricity) and the detection signal (light) are totally different, which is why ECL presents a lower background signal, resulting in higher sensitivity than electrochemical detection. Hence, it has been widely employed as analytical technique, in particular as a detection system in sensors and biosensors. Besides its high sensitivity, other advantages are the broad dynamic range; the simplicity of the analysis methodology (similar to conventional ELISA), the great flexibility, due to the high stability of luminophores; and the facility of combining with biological systems. Due to its combination of electrochemical and optical advantages, ECL has been widely used in the detection of biomolecules in a variety of samples, including plasma, whole blood, serum and cell supernatant. ECL has been also successfully used as detector [16] of high-performance liquid chromatography (HPLC) [17], flow injection analysis (FIA) [18], micro total analysis (μTAS) [19] and capillary electrophoresis [20]. The great success of ECL immunosensor due to the minimal background signals, better specificity and higher sensitivity is increasing the use of meso scale discovery (MSD) ECL assays [21]. MSD assays work on the same principle as ELISA but use ECL as the detection method. Moreover, it shows the ability to measure multiple analytes in a single well, having also the possibilities of analysis high amounts of samples in short times thanks to the well-plate configuration.

Developing ECL biosensors is now a hot topic that is increasing the interest of the analytical chemistry community. In addition to the advantages already mentioned, the scalable instrumentation required and the possibility of combining them successfully with screen-printed electrodes (SPEs) technologies are promoting the development of new devices, most of them adaptable to the new trend of point of care (POC) systems.

SPEs’ manufacturing process is characterized by the fast and easy mass production of reproducible electrochemical platforms, which offer true potential for application in the field of sensor [22]. SPEs are disposable electrochemical devices that avoid tedious pre-treatment steps. Hence, they are suitable for on-site and real-time sensing [23]. Moreover, the use of screen-printing technology also offers other interesting advantages, such as electrodes with different spatial distributions, appropriated for miniaturized devices [24] and the use of small sample volumes. Thus, SPEs have been widely employed in enzymatic biosensor [25,26,27], immunosensors [28,29,30,31,32] and DNA sensor fabrication [26,30,33,34,35,36,37,38].

The development of new miniaturized instruments integrating SPE and ECL has expanded the use of the technique to routine analysis. Among these, the *SpectroECL* and *µStat ECL,* commercial instruments from Metrohm-Dropsens, stand out for their ease of use. These instruments are small, portable and the SPEs are perfectly integrated into the device, being a convenient and a great affordable alternative.

In the last two decades, nanomaterials have burst into the field of ECL biosensors. Combining the bioselectivity and specificity of the biorecognition element with the numerous advantageous chemical and physical properties of nanomaterials has allowed the development of a whole new subset of sensitive biosensor devices. Therefore, great effort has been made in the development of new synthetic strategies to prepare a great variety of nanomaterials with highly controllable size, shape, surface charge and physicochemical properties [39]. In addition, functionalized nanomaterials show excellent properties for interfacing biological recognition events with electronic signal transduction in the design of a new generation of devices that exhibit new functionalities [40]. The appropriate use of nanomaterials clearly enhances the analytical properties of biosensors, increasing the sensitivity and lowering the detection limits in several orders of magnitudes.

A well-known advantage of nanomaterials is they provide a large effective surface area, which already enables the immobilization of higher amounts of bioreceptor units on the electrode surface [41]. They can also be used as a support of the biorecognition layer. Nanomaterials and biomolecules are in the same size range, facilitating the formation of hybrids with synergistic properties. They can act as labels of the recognition event or as catalyst for a reaction, as luminophore or as the energy acceptor to lead to an effective ECL resonance energy transfer (ECL-RET) [42]. Hence, the efficiency of ECL biosensors will be increased due to the use of different nanomaterials, such as graphene, carbon dots, quantum dots, metal nanoclusters, etc. combined with the recognition element and electrode modification strategy more appropriate [43].

ECL sensor and biosensor miniaturization is another area with great growth expectation in combination with microfluidic platforms or microarray technology and nanomaterials. All these areas together will improve the development of ultra-sensitive lab-on-a-chip systems for a variety of biosensing devices applied in clinical, food and environmental industries.

This review focuses on the use of screen-printed electrodes combined with nanomaterials in ECL biosensors, specifically how the biorecognition elements are immobilized on SPE and how nanomaterials are incorporated into the biosensor devices. ECL immunosensors, ECL enzyme-based biosensors and ECL DNA-based biosensors are discussed.

We believe this review will provide a base of knowledge in the development of ECL biosensor devices based on disposable SPEs, which promise selective and sensitive determinations of analytes in a wide range of samples, thanks to high affinity/biocatalytic interactions of the analytes with bioreceptors and the great advantages of ECL analysis methods. Thus, these devices will be marketed and widely used as rapid sensing and POC systems.

### Screen Printed Electrodes (SPEs)

During the last decades, screen-printing technology, highly developed in microelectronics applications, has offered extraordinarily high-volume production of extremely cheap and highly reproducible and reliable single-use electrodes [44]. SPEs have been traditionally produced by printing different inks on plastic or ceramic substrates. The composition of the various inks used for SPEs manufacturing are of great importance for the electrochemical properties of the electrodes. Carbon materials and graphite derivate in concrete are preferred in the ink composition due to their simple technological processing and low-cost. However, the use of metal particles in different ink compositions is also widespread in SPE fabrication. Among the most used metals, we can highlight gold, silver and platinum. Furthermore, materials such as Indium Tin Oxide (ITO) and poly(3,4-ethylenedioxythiophene) (PEDOT), which have the great advantages of being optically transparent for visible range, have also been employed to manufacture SPEs.

The great versatility of SPEs is due to the wide range of strategies in which the working electrode surface can be modified. Apart from the different ink composition, including the possibility of using a great variety of compounds to modify it with the aim of controlling its electrochemical behavior, the uncountable different strategies to modify SPE surface have allowed the SPEs employment in a multitude of sensors and biosensors. The great biocompatibility of some of the ink materials together with the successive modification by different strategies in a layer-by-layer system have been decisive for the appearance of a huge number of electrochemical and ECL biosensors. Another key factor of SPE modification was the appearance of a great variety of nanomaterials. This fact can be considered a great revolution in the field of electrodes modification due to the numerous and advantageous chemical and physical properties of nanomaterials. Nanomaterials have also demonstrated extraordinary properties and new interaction ways with biological elements and have been widely employed in biosensor development.

The disposable nature of SPEs is related with fundamental properties such as portability, low-cost, ease of use and mass production [45]. During the last decades, it has been a strong trend for disposables to be built with low-polluting materials, to minimize the amount of waste during the manufacture procedure, and the entire device must be recyclable for new uses [45]. In this way, paper is the most used material for the development of disposable sensors. It is a suitable material with high compatibility with inkjet [46] and screen printing techniques [44]. Moreover, it can be successfully combined with different surface modifiers such as polymers [47], nanomaterials [48], redox mediators [49,50] and biological element [51]. Different techniques such as photolithography, wax printing and chemical vapor-phase deposition have been successfully applied to transform paper platform into SPE. Apart from these sophisticated techniques for paper electrode modification, the creation of pencil drawn electrodes (PDEs) for electroanalytical applications has also been demonstrated, as it has previously been demonstrated in platforms such as paper [52], polyester [53] and PVC [54]. The rich surface chemistry of cellulose-based materials is a great advantage during the immobilization of bio-receptors by physisorption [55], bio-affinity interaction, by covalent bond due to a variety of chemical reactions (active esterification, maleimide cycloaddition, click chemistry, diazonium chemistry, etc.) [56]. All these excellent properties of the paper-based electrodes are making them a really useful option for competitive future electrochemical and ECL applications.

The great ambition to be able to monitor and measure in real time the presence of different analytes in different fields such as the environment, crops, industrial production chains, food production lines as well as in the human body itself have led to a great development of SPEs. The great diversity of different supports materials for SPEs has allowed its application in all the mentioned fields, each with particular requirements that can be supplied by the combination of different electrode supports and different inks for working electrode impression. A great advantage of some used materials as SPE supports is its flexibility and adaptability, which has been a great advance in the development of wearable devices [57]. The point-of-care diagnosis (POC) has been revolutionized by the development of these SPEs as a consequence of their disposability. All these great advantages make SPEs real competitors compared with conventional electrodes; consequently, they have been extensively employed in electrochemical and ECL biosensor development.

## 2. ECL Immunosensors

ECL has also become an important and powerful analytical technique in the field of immunosensors. Significant features such as high sensitivity, high reproducibility, versatility, simple optical technology and low background signal make ECL a good option for immunosensor development. In particular, SPEs are employed as electrochemical platforms because of their advantages in terms of low cost and rapid mass production, portability, high sensitivity, low sample volume requirement and easy handling [29]. The main drawbacks of the use of SPEs are the difficult of electrode surface regeneration and their reusability. These disadvantages are usually assumable if the immunosensor platform production is affordable.

During the last years, huge effort has been done to obtain higher sensitivity and develop new applications of ECL immunosensors based on different strategies. The complexity of the luminescence mechanism, which involves mass transport and electron transfer dynamics of abundant radical intermediates electrogenerated on the surface carry to low ECL efficiency [13,58]. Therefore, numerous ECL systems have been designed to enhance the ECL emission of luminophore through co-reactant pathways.

Different immunosensor strategies have been followed using ECL developments. Among the most common strategies sandwich-type immunosensor are widely used. Moreover, in the literature, there are many examples including label-free (direct or indirect immunosensor) and competitive immunosensors. The immunosensor classification is based on the most common immunosensors types reported on the literature for ECL immunosensors:

- The label-free configuration is usually the simplest. They are based on the immobilization of a capture antibody over the electrode surface followed by the specific analyte recognition by the capture specific antibody, resulting in the retention of the analyte over the electrode surface. This simplest change over the electrode surface affects the ECL signal, generating an increase of the ECL emission if the analyte acts as co-reactant of the ECL used system or in the case that the diffusion of luminophore or co-reactant will be favored as a consequence of less steric hindrance.
- Competitive configuration is based on the competition of labeled and unlabeled analytes for a limited number of antibody binding sites [59]. Only one antibody is used in a competitive configuration, which is usually attached to the electrode surface. Labeled analytes are usually modified with luminophore species, obtaining ECL signals which decrease with the higher concentration of the analyte of interest, as a consequence of fewer labeled analytes attached over the electrode surface.
- Sandwich type configuration is one of the most widely used on ECL immunosensor. It is based on the immobilization of a capture-specific antibody on the electrode surface (or over magnetic beads in some cases). After that, the specific antigen (analyte) is bonded over the capture antibody. Then, a secondary labeled antibody (detection antibody) reacts with the previous immobilized analyte. Considering the different labels attached to secondary antibodies, different configurations of sandwich-type ECL immunosensors have been developed:
  ○ The traditional sandwich type immunosensor is based on the linkage to the secondary antibody of a luminophore molecule or a nanomaterial, which acts as luminophore itself or is used as support to attach a great number of luminophore molecules.
  ○ The quench-type electrochemiluminescence immunosensor uses a secondary antibody linked to an element capable of generating resonance energy transfer (RET) phenomena of the emitting specie [60,61]. The element capable of quenching the ECL emission is usually a nanomaterial or a composite nanomaterial, decreasing the ECL emission when higher amounts of analytes are present in the sample.
  ○ A kind of sandwich-type ECL immunosensor that has recently appeared is named Faraday-cage-type electrochemiluminescence immunosensor [62,63]. The more important difference from the traditional sandwich-type immunosensor is that faraday-cage-type immunosensor uses a conductive two-dimensional nanomaterial (e.g., graphene, among others) simultaneously coated with a luminophore and a recognition component such as detection antibody, which could directly overlap on the electrode surface. In that configuration, electrons could flow freely from the working electrode surface to the detection element, extending the outer Helmholtz plane (OHP) of the electrode. This strategy allows the two-dimensional nanomaterial coated by thousands of luminophore molecules, being all of them electrochemically “effective” and really close to the working electrode surface.

### 2.1. [Ru(bpy)_3_]^2+^ ECL Systems

Many ECL immunosensors with SPEs as electrochemical platform use ECL complex Tris(bipyridine) ruthenium (II) [Ru(bpy)_3_]^2+^, together with different co-reactants. The ECL signals are controlled by the greater or lesser steric hindrance of the ECL probe to reach the SPE surface. Usually, the antigen–antibody union generates a stiffer structure that causes a slight steric hindrance allowing the complex [Ru(bpy)_3_]^2+^ and the co-reactant to reach the SPE surface. As result, a higher ECL signal is obtained. In this sense, Rizwan et al. [64] have reported a label-free immunosensor for beta 2-microglobulin based on a composite material made of CdSe quantum dots and gold nanoparticles (AuNPs) decorated carbon nano-onions. The nanocomposite is employed together with the widely used co-reactant tri-n-propylamine (TPrA). CdSe quantum dots acts as an additional co-reactant, while gold nanoparticles decorated carbon nano-onions amplified the ECL signals due to their high conductivity. This immunosensor shows a really wide linear range from 1 fg/mL to 100 ng/mL.

In a completely opposite approach, ECL immunosensor response is based on the higher steric hindrance caused by addition of a secondary specific antibody in a sandwich configuration. This high protein electrode coating prevents the ECL probe or the co-reactant to reach the SPE, giving rise to the ECL signal decrease as the antigen concentration increases. Breast cancer antigen 15-3 (CA15-3) is determined from the ECL signal decrease on increasing the biomarker concentration [65].

The complex [Ru(bpy)_3_]^2+^ can be either in solution or immobilized on the electrode surface. Pu et al. [66] reported a composite nanomaterial including [Ru(bpy)_3_]^2+^ onto Pt nanoparticles, which is used to modify by dropcasting the working electrode surface. The developed immunosensor is specific to *Clostridium Perfringens*, an anaerobic bacillus that often causes gas gangrene. Anti-*C. Perfringens* antibody is immobilized on the working electrode surface, acting as capture antibody. As detection label, a composite nanomaterial based on type A antibody anti-*C. Perfringens* and Glucose dehydrogenase is used, being both of them adsorbed over ferrite nanoparticles covered by a gold layer. The ECL signal measurement is carried out in a solution containing gluconolactone and nicotinamide adenine dinucleotide (NAD), which is reduced to nicotinamide adenine dinucleotide hydride (NADH) and acts as co-reactant of [Ru(bpy)_3_]^2+^, generating ECL signals directly proportional to *C. perfringens* concentration. A great advantage of this ECL immunosensor is the analysis only requires 1 h, while other methods used for *C. perfringens* determination such as anaerobic culture (three days), real time PCR (2 h) or gel electrophoresis with fluorescent labels for DNA hybridization detection (9 h) are time consuming.

**Figure 2:**
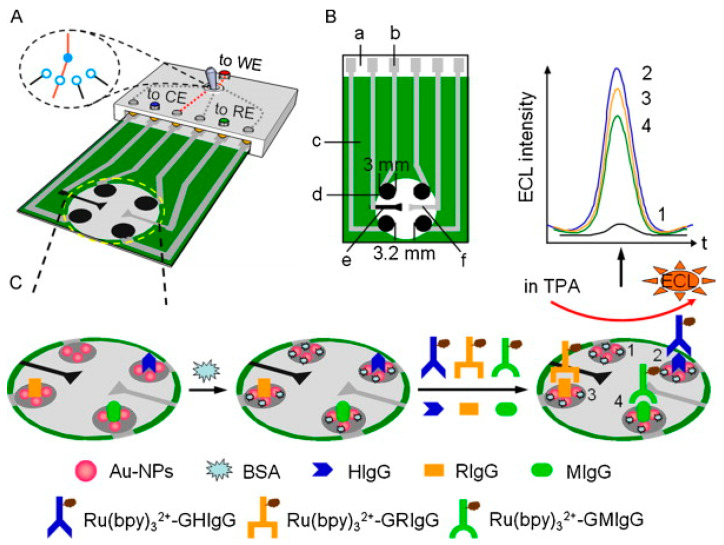
Schematic drawings of: (**A**) the immunosensor array for goat, rabbit and human immunoglobulins detection reported by Li et al. [67], held and controlled by a single-pore-four-throw switch; (**B**) SPE array containing four working electrodes, one reference electrode and one counter; and (**C**) immunosensor array preparation and immunoassay procedure. (**a**) Polyethylene terephthalate; (**b**) Ag conductive channels; (**c**) insulating layer; (**d**) carbon working electrode (WE); (**e**) carbon counter electrode (CE); and (**f**) Ag/AgCl reference electrode (RE). Reproduced and adapted with permission of Elsevier.

In other strategies, [Ru(bpy)_3_]^2+^ is covalently bounded to the detection antibody. Li, C. et al. [67], synthesized Ruthenium bis(2,2-bipyridine) (2,2-bipyridine-4,4-dicarboxylic acid) N-hydroxysuccinimide ester ([Ru(bpy)_3_]^2+^-NHS ester) that is bounded to different detection antibodies, through the reaction of N-hydroxysuccinimide with the carboxylic groups of the antibody structure, to develop a multiplexed immunoassay. Goat, rabbit and human immunoglobulins are attached to three of four electrodes on a screen-printed electrochemical array, maintaining one unmodified electrode as a control. In a competitive immunoassay, a solution containing the sample (consisting on different amounts of goat, rabbit and human immunoglobulins) and the different anti-immunoglobulins antibodies modified with [Ru(bpy)_3_]^2+^ is dropped over the four working electrodes, letting the specific recognition reaction takes place. Figure 2 summarizes the developed system. The ECL intensity decreases on increasing the concentration of immunoglobulins in the serum samples.

In a similar strategy, [Ru(bpy)_3_]^2+^ is linked to a streptavidin derivative to design an immunosensor for celiac disease diagnosis [68]. The immunosensor is specific to the celiac disease biomarker antitransglutaminase type-2 antibodies (anti-tTG). It consists of an ECL platform based on membrane-templated gold nanoelectrode ensembles (NEEs). In this work, an innovative sensing strategy is followed, based on the spatially separation of the initial electrochemical reaction and the immobilized biomolecules’ location, where ECL light is generated. As a recognition element, the authors employed tissue transglutaminase (tTG) immobilized on the polycarbonate (PC) surface of the track-etched templating membrane. Transglutaminase antibody (anti-tTG) is retained on the surface, thanks to its reaction with tTG. This mechanism permits the immobilization of streptavidin-modified ruthenium complex luminophore via reaction with an appropriate biotinylated detection antibody. An oxidizing potential is applied when the electrodes are immersed in a tri-n-propylamine (TPrA) solution. The electrochemical oxidation of TPrA generates TPrA*^+^ and TPrA* radicals that react with the [Ru(bpy)_3_]^2+^ ECL label, resulting in a great ECL signal, which is proportional to anti-tTG concentration with a linearity range between 1.5 ng/mL and 10 μg/mL and a detection limit of 0.5 ng/mL. A particular advantage of this strategy is that the ECL emission is obtained by applying a potential of 0.88 V versus Ag/AgCl, which is about 0.3 V lower than in the case when ECL is initiated by the electrochemical oxidation of [Ru(bpy)_3_]^2+^.

The use of nanomaterials to support [Ru(bpy)_3_]^2+^ is also widespread. Yang, H. et al. [52] developed an immunosensor to determine carbohydrate antigen 199 (CA199), a well-known tumor marker for early diagnosis of colon and pancreas cancer. AuNPs covered by [Ru(bpy)_3_]^2+^, just by adsorption, are linked to the detection antibody and used as label for ECL detection. The ECL signal is proportional to the logarithm of the CA199 concentration. An interesting feature of this immunosensor is the use of hand-drawn written pen-on-paper electrochemical platforms, which make it an affordable and easy way to prepare the device.

Among nanomaterials employed to support [Ru(bpy)_3_]^2+^ as ECL label, silica nanoparticles are very common. The immunosensor for whole cells of *Francisella tularensis* detection (responsible for tularemia infectious disease) is an illustrative example [69]. In this work, silica-encapsulated [Ru(bpy)_3_]^2+^ nanoparticles are synthesized and coated with polydiallyldimethylammonium. The detection antibody is covalently bounded through the carboxylic groups provided by addition of polyacrylic acid. ECL signals are directly proportional to *Francisella tularensis* concentration. This immunosensor shows a highly competitive result, with a limit of detection of 70 CFU/mL, whereas the reported limit of detection of the ELISA kit to determine this microbiological agent is 10^3^ CFU/mL. Silica nanoparticles coated with [Ru(bpy)_3_]^2+^ bounded to the detection antibody have also been used in an immunoarray for different prostate cancer biomarkers (prostate specific antigen (PSA), prostate specific membrane antigen (PSMA) and platelet factor-4 (PF-4)) [70].

Nanocomposite materials using a combination of silica and gold nanoparticles bounded to [Ru(bpy)_3_]^2+^ have also been employed in different immunosensors, e.g., one developed for cell cancer detection [71] and the alpha-fetoprotein immunosensor based on a magnetic nanocomposite material developed by Gan, N. et al. [72] The advantages of using SiO_2_ is they can load a large amount of [Ru(bpy)_3_]^2+^ while gold particles could provide large active surface to incorporate large amount of biological recognition element.

**Figure 3:**
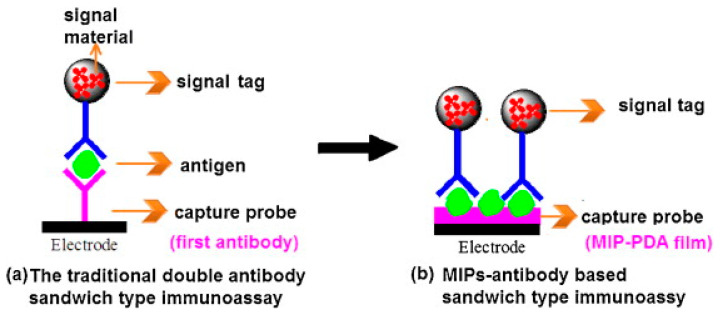
(**a**) The traditional double antibody sandwich type immunoassay; and (**b**) MIPs-antibody based sandwich type immunoassay developed by Feng, X. et al. [74]. Reproduced and adapted with permission of Elsevier.

The use of a composite nanomaterial based on [Ru(bpy)_3_]^2+^@silica@AuNPs conjugated with the detection antibody has also been developed in combination with magnetic molecularly imprinted polymers (MMIP) as capture probes. Zhou, J. et al. [73] followed this strategy to construct a “single antibody sandwich” type immunosensor capable of ultratrace detection of hemoglobin. A similar strategy using molecularly imprinted polymers (MIP) instead of an antibody as capture probe has been used for carcinoembryonic antigen (CEA) and carbohydrate antigen-199 (CA199) immunosensors (Figure 3). As signal tag, Feng, X. et al. [74] employed a nanocomposite material based on [Ru(bpy)_3_]^2+^-silica@poly-l-lysine-AuNPs linked to the detection antibody.

Carbon nanomaterials are very common in the composite material used to prepare ECL immunosensor labels. Hou, J. et al. [75] employed a composite nanomaterial, based on multiwall carbon nanotubes, to adsorb the ECL probe [Ru(bpy)_3_]^2+^. The composite nanomaterial is them cover with AuNPs, which are used to link the detection antibody by adsorption. This new composite nanomaterial is used as ECL tag, amplifying the ECL signal and acting as immobilization substrates for detection antibody. Another example of the use of carbon nanomaterials is the mouse IgG (MIgG) immunosensor developed by Zhou, H. et al. [76]. The capture antibody (anti-MIgG) modified with the ECL probe [Ru(bpy)_3_]^2+^ is covalently linked to the working electrode. The ECL signal is not affected by the reaction between the target analyte (MIgG) and the capture antibody; thus, they developed a competitive immunosensor. The authors prepared amorphous carbon nanoparticles (ACNPs), which have a quenching effect on the ECL signal of [Ru(bpy)_3_]^2+^. These ACNPs are modified with the MIgG (ACNPs-MIgG), so the unoccupied capture antibodies immobilized on the working electrode after incubation with the sample, react with ACNPs-MIgG, generating a decrease of the ECL signal when higher quantity of ACNPs-MIgG are retained. Therefore, the ECL signal is directly proportional to the concentration of MIgG at the sample. The method shows a linear range of 0.50–400 ng/mL and a detection limit of 0.35 ng/mL.

[Ru(bpy)_3_]^2+^ is widely used for anodic ECL, but some examples have demonstrated that, in combination with S_2_O_8_^2−^, it can also be used for cathodic ECL. Fang, D. et al. [77] developed an immunosensor for thyroglobulin, an important biomarker for postoperative tumor recurrence or persistence in patients with thyroid cancer. The immunosensor is based on a TiO_2_ nanodots modified electrode, where the capture antibody is immobilized. A bioconjugate formed by MXenes layers covered by [Ru(bpy)_3_]^2+^ and ZnO quantum dots, modified with the detection antibody are used as recognition element. The ECL signal is proportional to the logarithm of biomarker concentration. A specific characteristic of this immunosensor is the photothermal ECL amplification, which provides a low limit of detection and wide linear range. The amplification is due to ZnO quantum dots, which raises the electrode surface temperature by converting laser energy into heat.

### 2.2. Luminol ECL Systems

Luminol (2,3-aminophthalhydrazide) is a widely used ECL luminophore on immunosensor development. It has been used for anodic ECL together with oxidant species as co-reactant such as H_2_O_2_, O_2_, C_2_O_4_^2−^, etc. Luminol and its co-reactant can be used in solution, as in the case of the human immunodeficiency virus (HIV) type 1 antibody immunosensor developed by Zhou, J. et al. [78]. The immunosensor is based on the use of MMIP as capture probes. Human immunoglobulin G (HIgG) is used as the template for the MIP, as it exhibits the same antibody Fc region but a different Fab region as the anti-HIV-1. MMIP recognize the anti-HIV-1 present in the sample. The conjugated horseradish peroxidase-antigen (HRP-HIV-1) is used as label. HRP catalyze the oxidation of luminol in the presence of H_2_O_2_, generating an increase of ECL signal when higher amounts of anti-HIV-1 are retained on the MMIP particles. To generate the electrochemical required process, carbon SPEs are used together with a magnet under the working electrode, in order to transfer the modified MMIP to the electrode surface. A linear range of the anti-HIV-1 dilution ratio (standard positive serum) was obtained from 1:20,000 to 1:50, with a detection limit of 1:60,000. The developed method provides a low-cost, simple and sensitive way for the early diagnosis of HIV infection.

**Figure 4:**
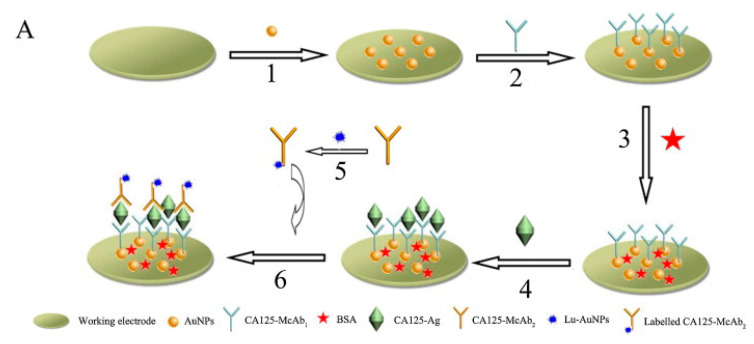
Schematic representation of the fabrication of the ECL CA125 immunosensor and assay procedure described by Wang, S. et al. [80]. Reproduced and adapted with permission of Elsevier.

Luminol can also be covalently attached to detection antibodies. In the alpha-fetoprotein (AFP) immunosensor developed by Su, M. et al. [79], macroporous Au-paper working electrodes (Au-PWE) are modified with the capture antibody (anti-AFP), which traps the breast cancer cells (MCF-7) through the AFP molecules of their membrane. The labeled detection antibody (anti-AFP-Luminol) interacts with the trapped MCF-7 cells. The anodic ECL signal obtained, using H_2_O_2_ as co-reactant, is directly proportional to the logarithm concentration of AFP present over MCF-7 surface in the range of 0.01 until 200 ng/mL. In another study, carcinoma antigen 125 (CA125) is detected in clinical serum samples using carbon ink over previously wax-patterned cellulose paper to fabricate disposable SPEs [80]. As illustrated in Figure 4, after electrode modification with adsorbed gold nanoparticles (AuNPs), L-cysteine is chemisorbed through the thiol group. 1-ethyl-3-(3-dimethylaminopropyl) carbodiimide/N-hydroxysuccinimide (EDC/NHS) activated carboxylic groups of chemisorbed L-cysteine allow covalent link of anti-CA125 capture antibody through the reaction of amino groups from Fc antibody region with the activated carboxylic acid of L-cysteine. As ECL label, anti-CA125 secondary antibodies are modified with covalently linked luminol-AuNPs [81]. The labeled antibody is retained by the CA125 antigen previously attached over the working electrode surface. The ECL measurement is carried out using a cathodic scan and the signal increases proportionally to the logarithm of CA125 concentration.

A label-free ECL immunosensor for transferrin (TRF) has been developed using luminol-reduced gold nanoparticles (Lu-Re-AuNPs) modified carbon SPE [82]. Lu-Re-AuNPs composite is prepared in a chitosan polymeric film. AuNPs are used to adsorb Anti-TRF antibodies. Finally, the immunosensor is incubated with the sample containing TRF. The ECL signal is measured in anodic scan, using a carbonate solution buffer containing as co-reactant H_2_O_2_. The ECL signal decreases linearly with TRF concentration, as nonconductive proteins (as TRF) may interrupt the interfacial electron transfer and hinder the diffusion of the electrochemically active molecules. This method shows better sensitivity than other label-free immunoassays, such as immune precipitation and immune turbidity. It is also easily manipulated, affordable and the recognition activity of the immobilized antibody is highly stable.

### 2.3. S_2_O_8_^2−^ ECL Systems

S_2_O_8_^2−^ is the most used co-reactant employed on immunosensor development together with semiconductor nanomaterials responsible of ECL emission. In this case, the ECL signal is obtained at the cathodic scan. The S_2_O_8_^2−^ is reduced to SO_4_*^−^ and SO_4_^2−^; SO_4_*^−^ reacts with the excited form of the semiconductor nanomaterial, which consequently falls down to the ground state generating the ECL signal. S_2_O_8_^2−^ is usually in the solution, while the semiconductor material can be attached to the electrochemical platform or be part of the ECL label used as detection element.

Accordingly, Wu et al. developed a label-free immunosensor for determination of the tumor marker carbohydrate antigen 125 (CA125) [83]. It is based on S_2_O_8_^2−^ as co-reactant and the semiconductor graphite-like carbon nitride (g-C_3_N_4_) modified with carboxylic groups and immobilized on the electrochemical platform. Aminated Fe_3_O_4_ nanoparticles and anti-CA125 (capture antibody) are linked to g-C_3_N_4_ through the reaction between activated carboxylic groups of g-C_3_N_4_ and amine groups of nanoparticles and antibody. As authors explained that carboxylated g-C_3_N_4_ transfers electrons from its conduction band to Fe_3_O_4_ nanoparticles, preventing carboxylated g-C_3_N_4_ from electrochemical degradation and simultaneously catalyzing the reduction of S_2_O_8_^2−^ into SO_4_*^−^, which leads to the enhancement of ECL emission of g-C_3_N_4_.

**Figure 5:**
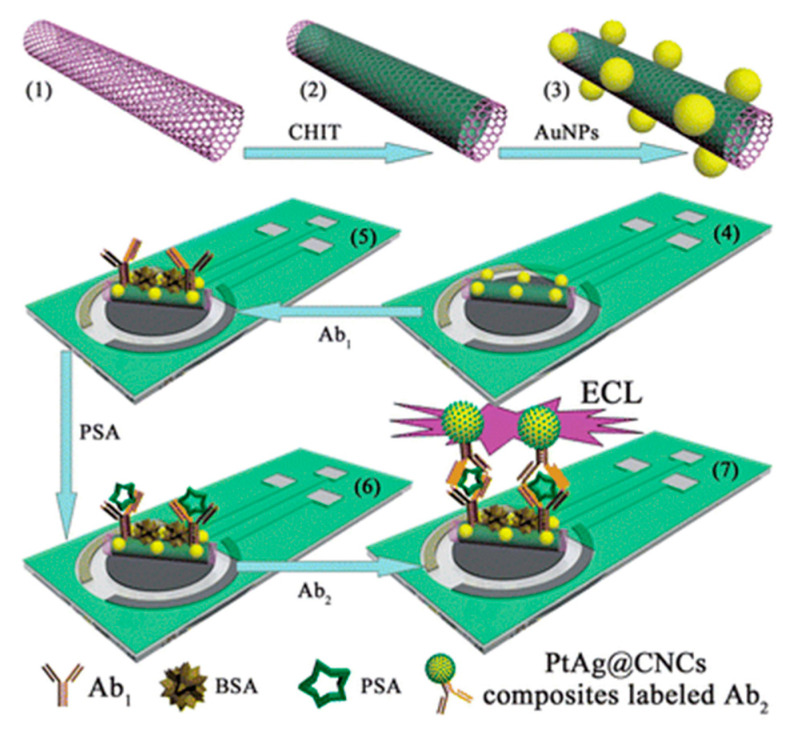
Schematic representation of the fabrication of the ECL immunosensor for PSA, developed by Zhang, M. et al. [84]: (**1**) CNT; (**2**) CNT-CHIT; (**3**) CNT-CHIT/AuNPs composite; (**4**) CNT-CHIT/AuNPs composite modified SPCEs; (**5**) after immobilization of Ab_1_; (**6**) capture with PSA; and (**7**) immobilization with the PtAg@CNCs composites labeled Ab_2_. Reproduced and adapted with permission of Royal Society of Chemistry.

Other strategies using S_2_O_8_^2−^ as co-reactant are based on the use of semiconductor nanomaterials as label of the recognition event between the antigen and a detection antibody. Zhang, M. et al. [84] used semiconductor carbon nanocrystals (CNCs) as ECL transmitter for a PSA immunosensor (Figure 5). CNCs are also modified with PtAg alloy, generating a composite nanomaterial (PtAg@CNCs), which is linked with the detection antibody (anti-PSA). The resulting ECL label reacts with the PSA antigen, previously attached by the capture antibody to the working electrode. In a first step, carbon SPE is modified with carbon nanotubes covered with chitosan polymer and AuNPs (CNT-CHIT/AuNPs composite). The capture antibody is immobilized onto the CNT-CHIT/AuNPs. In a sandwich immunosensor configuration, S_2_O_8_^2−^ is electroreduced due to the electrocatalytic activity of PtAg alloy, generating SO_4_*^−^ radicals that react with CNCs electrochemically excited by electroreduction, transmitting an ECL signal directly proportional to the PSA concentration logarithm.

Quantum dots have also been employed as ECL labels as they can behave as ECL transmitter, due to their semiconductor properties. In this way, CdTe quantum dots supported on porous silver nanoparticles (QDs/AgNPs) are used as ECL transmitter together with the co-reactant S_2_O_8_^2−^ [85]. Silver nanoparticles act as S_2_O_8_^2−^ reduction electrocatalyst, amplifying the ECL signal of the immunosensor designed for the determination of a tumor marker, carcinoembryonic antigen (CEA). The detection antibody (anti carcinoembryonic antigen) is covalently linked to QDs/AgNPs, acting as an ECL label of the sandwich-type immunosensor. It is interesting that the SPE electrochemical platform is based on a microfluidic origami device fabricated directly on wax-patterned cellulose paper, which make it a really affordable system. It is additionally modified with graphene nanosheets to covalent bond the capture antibody. The ECL signal is proportional to the CEA concentration logarithm.

Another similar development described by Zhang, Y. et al. is based on the use of carbon SPE modified with graphene nanosheets previously modified with silver and gold nanoparticles [86]. This composite nanomaterial acts as support of the capture antibody specific to CA 125 and facilitates the electron transfer. CdTe quantum dots coated carbon microspheres (QD@CM) are used as ECL label. QD@CM composite are modified with the detection antibody using EDC/NHS. The bioconjugate QD@CM/Ab_2_ acts as ECL label of the sandwich-type immunosensor generating an ECL signal directly proportional to the CA 125 concentration logarithm. This tumor biomarker immunosensor has been applied to serum samples, obtaining results in agreement with those obtained with the reference method (commercially available Electrochemiluminescent Analyser ROCHE E601, Switzerland) and relative standard deviations about 2.84–7.31%.

The ECL behavior of semiconductor SnO_2_, with lower biotoxicity and better stability, has seldom been investigated. Ma, C. et al. [87] reported a carcinoembryonic antigen (CEA) immunosensor, where SnO_2_ nanocrystal (NC) is applied as a novel ECL signal reporter. Carbon SPEs are modified by dropcasting with nanoporous silver (obtained by dealloying method from Ag_23_Al_77_ alloy foils). The capture antibody is adsorbed on the resulting modified electrode to link CEA molecules present in the sample. Herein, SnO_2_ nanocrystals are combined with PtRu alloy, which enhances the ECL signal. For this porpoise, SnO_2_ is first functionalized with amino groups by reaction with (3-Aminopropyl) triethoxysilane and then bonded to PtRu alloy nanoparticles, being the amino groups coordinated with Pt atoms. Finally, the nanocomposite materials are functionalized with the detection antibody, by coordination of amino group of the antibody with the Pt atom. This nanocomposite material is used as ECL probe through the reaction of SO_4_*^−^ radicals generated with reduced SnO_2_ nanocrystals. Herein, the reduction of S_2_O_8_^2−^ to SO_4_*^−^ radicals is electrocatalyzed by PtRu alloy, increasing the ECL signal linearly with CEA concentration. The immunosensor has a limit of detection as low as 0.72 pg/mL, which is lower than of the other methods.

In a similar approach, ZnO quantum dots are employed for PSA determination [88]. Pt/AuNPs are previously electrodeposited on the carbon SPE. In a sandwich-type configuration, the capture antibody is attached to the Pt/AuNPs. As recognition label, the authors used a composite nanomaterial consisting on ZnO quantum dots synthesized on carbon nanotubes (ZnO@CNT) that is linked to the specific detection antibody. The ECL signal is obtained in presence of S_2_O_8_^2−^, which generates SO_4_*^−^ that reacts with reduced ZnO*^−^ emitting an ECL signal directly proportional to the concentration of PSA.

### 2.4. Other ECL System

**Figure 6:**
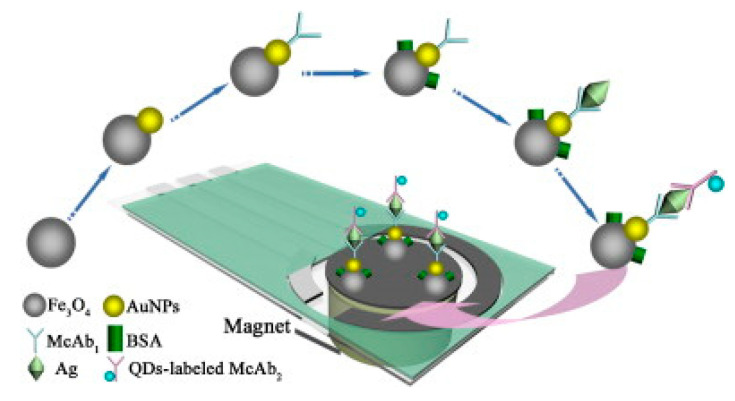
Schematic representation of the CEA ECL immunosensor developed by Wang, S. et al. [89]. Reproduced and adapted with permission of Elsevier.

Over recent years, besides the traditional ECL systems described above, new ECL systems based on nanomaterials have been used to develop immunosensors based on SPEs. Most of these alternative systems are based on the use of quantum dots as ECL emitters. Wang, S. et al. [89] developed a CEA immunosensor based on indium tin oxide (ITO) SPE. As shown in Figure 6, gold-coated magnetic iron nanoparticles (Fe@Au MNPs) are employed as solid support to CEA primary monoclonal antibody (CEA-McAb_1_). CdTe quantum dots (QDs) are used to label CEA-secondary antibodies (CEA-McAb_2_). By the help of a magnet, the sandwich-type immunoassay that takes place over the magnetic particles is transferred to the ITO SPE surface. The ECL signal is obtained during the anodic scan, when CdTe quantum dots are oxidized (CdTe(hole^+^)), being the electron rapidly transferred into an O_2_ molecule dissolved in the buffer. The oxygen radical generated (O_2_*^−^) react with CdTe quantum, generating CdTe(e^−^), which reacts with CdTe(hole^+^) emitting ECL. This ITO ECL device is an interesting alternative to ceramic- and polyethylene terephthalate (PET)-based carbon SPEs. ITO as transparent material improves the transmittance of ECL emission, enhancing the sensitivity.

A CEA immunosensor was also developed by Li, L. et al. [90] using in this case Au@Pt core-shell nanoparticles modified with graphene quantum dots (GQDs) as supporting for the detection antibody labeled with Glucose oxidase (GOx). The immunosensor is developed using nanoporous gold/chitosan modified paper electrode (NGC-PWE) as sensor platform. The ECL signal is based on the detection of H_2_O_2_ generated in the enzymatic reaction, which is instable and is transformed into (O_2_*^−^). This oxygen specie reacts with electrochemically oxidized graphene quantum dots (GQDs*^+^), generating excited GQDs* that emits an ECL signal when it returns to its fundamental state. The ECL intensity is proportional to the logarithm of CEA concentration. NGC-PWE improves electron transfer and allows the immobilization of a high number of antibodies. The loading of GQDs on Au@Pt gives rise to an amplified ECL signal and to the consequent improving of the sensitivity.

Carbon dots are new nanomaterials that are being used in ECL immunosensor. Liu, W. et al. [91] developed a CA125 immunosensor employing porous Ag-paper working electrode (Ag-PWE) to immobilize the capture antibody. In a sandwich-type configuration, the detection antibody is bounded to carbon nanodots modified nanoporous silica nanoparticles (MSNs). The ECL signal is obtained in the presence of triethylamine (TEA). At anodic scan, carbon nanodots and TEA are oxidized, generating radical species that react between them, generating excited carbon dots that emit ECL signal when they return to the ground state. The immunosensor present a wide linear range from 0.01 to 50 U/mL with a detection limit of 4.3 mU/mL.

Among others ECL luminophores, phenyleneethynylene derivatives because of its chemical stability and high efficiency of luminescence, have drawn intense attention in immunosensor field. A carcinoembryonic antigen (CEA) immunosensor has been developed using phenyleneethynylene derivatives ((4,4′-(2,5-dimethoxy-1,4-phenylene) bis(ethyne-2,1-diyl) dibenzoic acid (P-acid)) modified with mesoporous Pt–Ag alloy nanoparticles (P-acid/Pt–AgANPs) [92]. The working electrode is previously modified with graphene and a dense gold layer is formed through the growth of AuNPs on the graphene, which in turn will be beneficial to the sensitivity, stability and effective surface area of the SPE. Anti-CEA capture antibodies are immobilized onto the Au-Graphene layer. Finally, the P-acid/Pt–AgANPs composite is applied to the immobilization of a secondary antibody. According to sandwiched immunoreactions, once the sandwich-type immunosensor is incubated in the sample, CEA reacts with capture antibodies and the secondary antibody labeled with the composite nanomaterial, obtaining the ECL signal as the potential is scanned toward anodic values in a solution containing TEA. P-acid and TEA are oxidized during anodic scan, generating radical species that react between them. The ECL signal generated is directly proportional to the CEA concentration logarithm. This ECL immunosensor has two advantages: (1) the gold/graphene layer provides an effective antibody immobilization matrix with high stability and bioactivity; and (2) the P-acid/Pt–AgANPs system improves the ECL signal, obtaining better sensitivity.

ECL Immunosensors based on the use of SPE technology described above and their analytical properties are summarized in Table 1.

## 3. ECL Enzymatic Biosensors

Enzymes are proteins that catalyze chemical reactions in living organisms, behaving as highly selective catalysts that allow recognition of a wide variety of substrates of analytical interest. Therefore, enzymes immobilized on the electrode surfaces have been widely employed for the development of biosensors.

In general, enzyme immobilization methods are often classified in two broad categories: chemical bonding and physical retention. The covalent binding of enzymes to supports and cross-linking are the more prominent methods by chemical bonding [93]. The adsorption and entrapment of enzymes in porous substrates or the confinement of enzymes in semipermeable membranes are the main methods of immobilization by physical retention [94]. The most popular methods to build biosensors are cross-linking [95] and entrapment [96].

Among other factors, the choice of immobilization method must take into account the type of substrate that has to be processed and the conditions of the reaction. Another factor to consider in the case of ECL biosensors is the luminophore, which can be trapped together with the enzymes on the electrode surface or found in solution. Three main luminophores are used in enzyme biosensor such as [Ru(bpy)_3_]^2+^, luminol [97] and quantum dots (QDs) [98], being luminol the most used in oxidase enzyme biosensors. The light emission of luminol at the electrodes was first used in 1929 [99]. Luminol ECL is usually produced in alkaline solution with H_2_O_2_, when the anodic electrochemical oxidation of the electrogenerated excited monoanionic form of 3-aminophthalic acid occurs at a carbon or Pt electrode. The conditions under which ECL takes place are 0.6 V vs. saturated calomel electrode (SCE) and λ_ECL_ = 420 nm [100]. Because many oxide reductase enzymes can produce H_2_O_2_ during their catalytic activity, ECL enzyme biosensors can be carried out by coupling the light-emitting reaction produced by luminol with enzyme-catalyzed reactions that generate H_2_O_2_. Hence, luminol and its derivatives have been coupled with oxidase enzymes for the generation of ECL [97].

Clark and Lyons developed the first enzymatic biosensor in the 1960s, by immobilizing Glucose oxidase (GOx) on a platinum electrode to determine glucose. It revealed the possibility of enzyme immobilization on an electrode, resulting in enzyme-based amperometric biosensors that allow to detect electroactives species involved in the enzymatic reaction. Years later, Wilkinsont, J. S. developed an enzymatic ECL biosensor [101]. The glass waveguide is coated with indium tin oxide and modified with covalently attached GOx. The range of detection for glucose obtained was 0–10 mM with a detection limit of 0.3 mM. Since GOx is well-known and commercially available enzyme and diabetes mellitus is a non-infectious disease that has proliferated throughout the world, this enzyme is one of the most employed in biosensor development. Although it is not an acute disease, it can lead to complications such as kidney disease, blindness and foot disease or nerve damage. Tracking the diabetes related indices such as blood glucose concentration are very important to control and disease treatment. Therefore, enzymatic glucose biosensors have been a great deal of interest and new designs focus on the improvement of the sensitivity and selectivity have been described. ECL biosensors are one of the recent designs and GOx are the standard enzymes used for the majority of them. For this purpose, researchers have developed different strategies for the immobilization of GOx in the different supports. A sol–gel derived ceramic carbon composite electrode was used to develop a new type of biosensor based on luminol ECL for glucose [102]. The detection limit obtained was 8.16 mM. Guonan Chena et al. encapsulated GOx [103] in the film composed of Nafion and carbon nanotubes (CNTs). They reached an excellent electrocatalytic activity toward luminol, obtaining a detection limit of 2.0 µM glucose. Liu, L., et al. designed an ECL biosensor based on CdTe QDs and AuNPs [104]. The limit of detection for glucose was 5.28 μM. Other extremely sensitive glucose ECL biosensors were developed, where the authors utilized a membrane of poly(diallyldimethylammoniumchloride) doped with chitosan to immobilize GOx [105], obtaining a detection limit of 0.1 nM. Another combination of nanomaterials employed to fabricated a 3D platform was developed by electropolymerization of nickel (II) tetrasulfophthalocyanine (NiTSPc) on MWCNTs-modified glassy carbon electrode [106]. The detection limit obtained was 8.0 × 10^−8^ M. A high-performance three-dimensional 3D bio-platform of GOx adsorbed on AuNPs assembled polyaniline nanowires network was constructed. The ECL biosensor enabled the sensitive detection of glucose by an extremely low detection limit of 0.05 μM [107].

### ECL Enzymatic Biosensors Based on SPEs

**Figure 7:**
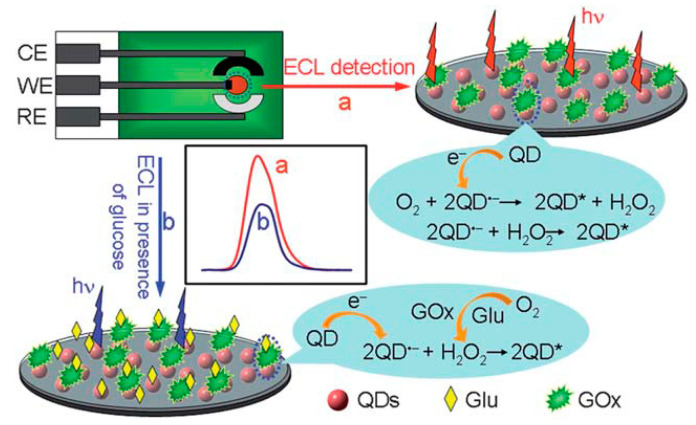
Schematic display of the ECL glucose biosensor developed by Cheng, L. et al. [108] employing bidentate-chelated CdTe QDs as emitters of ECL for glucose detection. Reproduced and adapted with permission of Royal Society of Chemistry.

As can be inferred from the approaches described above, several glucose ECL biosensors have been developed so far using conventional electrodes. SPEs are recently employed as they can provide additional advantages, in particular to fabricate disposable biosensors, making them important tools for the development of POC biomedical applications. In this sense, Cheng, L. et al. [108] developed a ECL biosensor immobilizing GOx and surface-unpassivated CdTe QDs on SPE (Figure 7). The glucose biosensor showed rapid response. The biosensor linear range extends from 0.8 to 100 mM, and the detection limit of it is around 0.3 mM. The detection of glucose in real human serum samples demonstrated acceptable sensitivity and selectivity. Yu, L. et al. [109] developed another disposable glucose ECL biosensor using Au and TiO_2_ nano-composite. GOx was cross-linked with BSA thanks to the use of glutaraldehyde, while Nafion is used for enzyme immobilization on the ITO surface. The biosensor has a linear range for the detection of glucose from 7.0 to 100 mM with a detection limit of 0.22 mM.

Uricase is an enzyme that catalyzes the oxidation of uric acid to 5-hydroxyisourate, which subsequently forms (S)-allantoin. It is involved in purine metabolism, urate degradation and (S)-allantoin formation. Its main use is the determination of uric acid in biological fluids. Hence, it is important for the development of disposable biosensors. Uricase has been immobilized for the fabrication of a uric acid ECL biosensor using two different strategies: trapped in a pyrrole matrix [110] or in a double-layer design of luminol as a copolymer with 3,3,5,5-tetramethylbenzidine (TBM) and chitosan on gold SPE [111]. The fabricated biosensor showed a sensitive response to uric acid with a low detection limit of 4.4 × 10^−7^ M and wide linear response (from 1.5 × 10^−6^ to 1.0 × 10^−4^ M). The biosensor turned out to be suitable for uric acid determination in 24-h urine samples compared to a reference procedure.

Another enzyme of interest is Lactate oxidase (LOx). Lactate is a chemical compound that plays important roles in various biochemical processes and is found in various biological fluids. Measurements of blood lactate concentrations are common in exercise and in clinical settings, as they can reveal information about the participant’s fitness. Blood lactate measurement is used as a marker of exercise intensity and training status. Therefore, methods with high sensitivity and good selectivity for the fast and dynamic lactate concentration response of lactate in human serum or other body fluids are urgently demanded. Lox-based biosensors for the determination of lactate in serum have been reported [112]. An ECL biosensor based on LOx and luminol with a higher sensitivity has been developed for the determination of this analyte in sweat [113]. Recently, Ballesta-Claver, J. et al. [114] developed a disposable lactate ECL biosensor based on LOx and luminol immobilized on a graphite SPE with Methocel membrane. The biosensor was applied to the analysis of lactate in saliva as an alternative procedure for obtaining the lactate level in a non-invasive way.

Other ECL enzyme biosensors have been developed so far, immobilizing different oxide reductase enzymes, such as cholesterol oxidase [115], glutamate oxidase [116], glycosylated hemoglobin [117], choline oxidase [118] and alcohol dehydrogenase [119], on conventional electrodes such as indium tin oxide glass, carbon and glassy carbon electrodes. Therefore, the possibility of developing SPE based disposable biosensors, benefiting from the advantages provided by this type of electrodes is open, as discussed in the Introduction.

**Figure 8:**
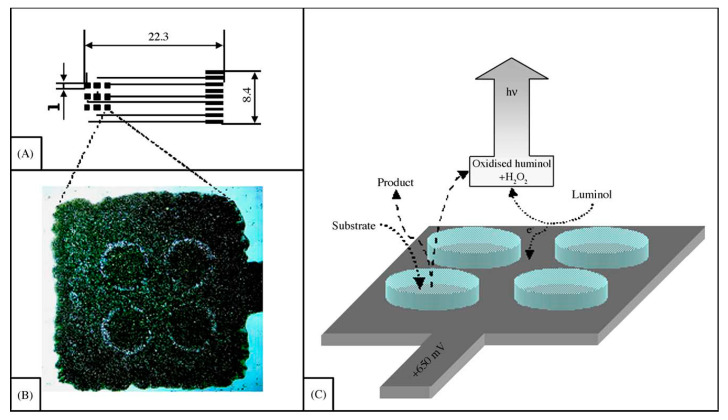
SPE multi-parametric glucose and lactate ECL Biosensor developed by Corgier, B.P. et al. [121]. (**A**) Schematic representation of the nine screen-printed array for ECL measurements. Size in mm. (**B**) Optical micrograph of the electrode surface (1 mm × 1 mm) supporting the four spots sensing layer. (**C**) Schematic representation of the sensing layer organization which is formed by a hybrid layer of photopolymer-Sepharose beads PVA-SbQ and reaction at a carbon electrode surface (1 mm × 1 mm). Reproduced and adapted with permission of Elsevier.

Besides, screen printing technology allows the fabrication of platforms with several working electrodes that share auxiliary and reference electrodes. Different enzymes can be immobilized on these devices to the development of selective and disposable biosensors for multiple analysis. Blum, L.J. et al. [120] were the first to describe an SPE multi-parametric ECL biosensor, based on ECL detection principles of enzymatically produced H_2_O_2_. Years later, GOx and LOx were trapped in PVA–SbQ (poly(vinyl alcohol), bearing styrylpyridinium groups photopolymer deposited on the surface of SPE array (Figure 8) [121]. The achieved multiple biosensor allowed the simultaneous detection and quantification of lactate and glucose with detection limits of 3 and 10 µM, respectively. These results demonstrate the analytical possibilities of SPE arrays to produce multi-parameter biosensors, based on ECL detection.

Enzymatic biosensors have many advantages compared to conventional analysis techniques, such as high specificity provided by the biological element, high sensitivity, short analysis times and the possibility of reusing the biological component, which, together with the advantages that ECL presents, makes enzyme ECL biosensors excellent candidates to continue investigating in this area.

In recent years, a huge boost has been observed in areas such as nanotechnology and nanoscience, being important the advances obtained in biomedicine [122], energy [123], catalysis [124] and electronic [125]. To a large extent, it has been due to the development and incorporation of new nanomaterials. This advance has also benefited Analytical Chemistry. In particular, in the field of ECL enzymatic biosensors, nanomaterials have provided significant improvements in the performance of these devices. By coupling nanomaterials and ECL, the signal-to-noise ratio increases compared with photoluminescence, since light scattering side effects are minimized. The development of new systems and strategies based on nanomaterials have allowed the determination of new analytes, even in very complex matrices [126]. Therefore, researchers have investigated the improvements that nanomaterials produce in ECL (bio)sensors.

In general, ECL enzymatic biosensors based on nanomaterials show a higher selectivity, sensitivity and stability. Furthermore, nanomaterials have provided improvements in the catalytic properties, by increasing the electronic transfer between the co-reactant and luminophores, as well as the conductivity, which lead to a high ECL response. In addition to the advantages explained above, nanomaterial have a great biocompatibility with enzymes, since they are in the same size range. Thus, they can form bioconjugates with synergistic properties. Finally, nanomaterials provide a large surface area, allowing the immobilization of a large quantity of enzyme molecules, as we shown in more detail below.

There are many nanomaterials used now for the development of enzymatic biosensors and their nature is broad, such as metallic nanoparticles of silver (AgNPs) [127] and gold (AuNPs) [128]; carbon nanomaterials as carbon nanodots (CNDs) [112], graphene [129] and carbon nanotubes (CNTs) [130]; and biochar [131]. Semiconductors such as quantum dots (QDs) [132] and silicates [133] are also investigated. All these nanomaterials can be deposited on the SPE and conventional electrodes surface for using as sensor platforms. There are different techniques for incorporating nanomaterials such as electrodeposition, as is the case of metallic nanoparticles [134] and CNDs [135], where more controlled coverage and reproducibility are obtained, through electrostatic interactions or formation of covalent bonds [93], click chemistry [136] with modified surfaces, electrospray deposition [137] and dropcasting [138], being the last one the most used, simplest and quickest way to prepare a sensing surface.

Some of the most used QDs in ECL biosensors are CdTe, CdSe, CdS and graphene quantum dots. These nanomaterials can act by themselves as luminophores generating an increase in the ECL signal, due to its good electronic, optical and photophysical properties. CdTe QDs have been used for the development of a disposable carbon SPE glucose biosensor [108]. In addition, these nanomaterials are widely applied in the design of other biosensors such as ECL immunosensors [132]. The QDs can also be managed together other semiconductors to gain synergetic system at the electrode where they can work as ECL enhancer since they act as efficient emitters of ECL [139]. ZnO is another kind of semiconducting nanomaterial that improves the ECL sensor response, which has been deposited on carbon and gold SPE for the determination of glyphosate [140] and taurine [141], respectively.

CNDs are water-soluble carbon nanomaterials with low toxicity and facile functionalization. Due to core-related quantum confinement effects of CNDs structure and edge effects near surfaces, they show electro-optical properties. These advantages make the CNDs suitable nanomaterial for the development of ECL biosensors, producing a response increase. CNDs have disclosed interesting features as nanomaterials for photoinduced electron transfer and ECL. In the last one, CNDs have demonstrated their ability to act as co-reactants [142], which is why this nanomaterial is widely used in the development of ECL biosensors [143].

AuNPs are characterized by, in addition to their stability and large surface area, excellent conductive properties, which allow improving the ECL responses by increasing their intensity. AuNPs can be trapped with hydrolyzed (3-aminopropyl) trimethoxysilane as linker to the electrode ITO surface [144] or deposited directly on the electrode surface [145]. AuNPs have not only provided a surface area increase but also formed a bioconjugate that improves the analytical performance of the ECL biosensor. Metal nanoparticles with the central core of Au and Ag have attracted great interest, due to their catalytic and plasmonic applications. Li, J. et al. [146] used them on glassy carbon electrodes to develop a ECL sensor for the detection of highly upregulated in liver cancer (HULC), obtaining extremely low detection limits.

The current trend in ECL biosensors is the combination of several nanomaterials, where the advantages of both nanomaterials are blended, leading to the formation of new materials with synergistic properties [147]. The use of these nanomaterials produces enhanced ECL signal intensity. Zhang, C. et al. [148] employed mixed nanomaterials as Au nanoclusters and CeO_2_ nanowires for the development of a biosensor. This ECL biosensor was based on the enzyme acetylcholinesterase. The use of CeO_2_ nanowires on glassy carbon electrodes improve the stability of Au nanoclusters. In this case, the authors proposed another mixture of nanomaterials, AgNPs were combined with carbon QDs obtaining a nanocomposite, that showed superb ECL activity in the development of an enzymatic glucose biosensor [149]. Du, X. et al. [150] proposed the use of CeO_2_ nanocrystallines assembled on the surface of graphene sheets doped with nitrogen (CeO_2_-NG) for the development of an ECL enzymatic biosensor based on cholesterol oxidase. The results display that the use of CeO_2_-NG nanocomposites facilitates the electrochemical redox process of co-reactant, improving the intensity of ECL. Numerous combinations of nanomaterials have been used until now and researchers continue to investigate new nanomaterials and their applications. The use of deposited nanomaterials, either on conventional electrodes or SPEs, produces considerable improvements in ECL biosensors. SPEs offer an additional advantage since they are disposable electrodes.

Enzymatic biosensors based on the use of SPE technology described above and their analytical properties are summarized in Table 2.

## 4. ECL DNA Biosensors

**Figure 9:**
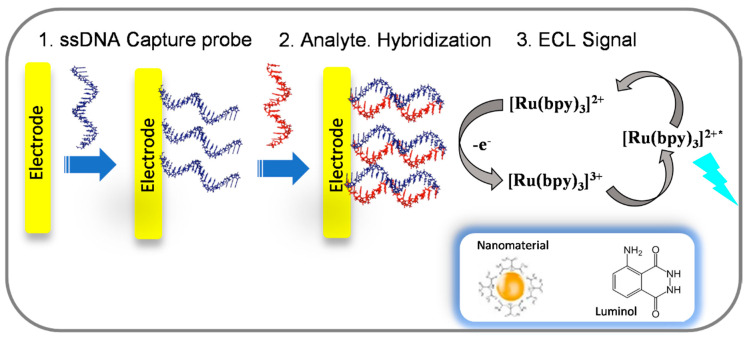
General scheme of ECL DNA Biosensor based on [Ru(bpy)_3_]^2+^.

DNA assays have many applications in different areas such as medical diagnostics, gene expression analysis, environmental investigations, biological warfare agent detection and pharmaceutical studies. DNA biosensors have been widely developed in different areas such as medicine and have brought enormous challenges to the early diagnosis of diseases [35,151,152,153]. In general, this kind of devices must have simplicity, fast response, good stability and high sensitivity to meet the requirements of clinical application and commercialization. In this sense, among the different transducing signal techniques, ECL is considered a promising one to improve the sensitivity and selectivity by lowering the background signal for DNA probe assays. ECL DNA biosensors use specific biological interactions to recognize and detect nucleic acids, producing a luminescent signal. An ECL DNA biosensor is a kind of biosensor in which a single-stranded DNA probe is immobilized on the electrode surface, which acts as a signal transducer of the hybridization event in an ECL signal by using different luminophores (Figure 9). Bard et al. [154,155] studied for the first time the ECL behavior of ruthenium and osmium pyridine complexes and DNA, paving the way for the ECL application in biological DNA analysis.

### 4.1. [Ru(bpy)_3_]^2+^, Luminol and Nanomaterials ECL Systems

As in the case of ECL immuno- or enzymatic biosensors described above, the generating ECL signals in ECL DNA biosensors are principally based on the use of luminophores as ruthenium complexes, luminol or nanomaterials. In the case of using ruthenium complexes as luminophore, a selective and highly sensitive detection of miRNA-21, based on the toehold-mediated strand displacement (TMSD) amplification with [Ru(phen)_3_]^2+^ loaded DNA nanoclews (NCs–[Ru(phen)_3_]^2+^) as signal tags, has recently been reported [156]. In this work, the stable DNA nanoclews, synthesized by a simple rolling circle amplification reaction, were employed with [Ru(phen)_3_]^2+^ efficiently as ECL signal tags to amplify the signals. The ECL intensity of the system act as a function of the logarithm values of microRNA-21 with a limit of detection of 0.65 fM. The strategy was further applied to detect miRNA-21 in complex samples (different cells containing the HEK-293, HeLa cells and MCF-7) and the result was consistent with the real-time quantitative reverse transcription PCR (qRT-PCR).

Concerning ECL DNA biosensors using luminol as luminophore, Gao, W. et al. [157] developed a new ECL DNA biosensor with high sensitivity and throughput to ECL imaging of nucleolin in a single HeLa cell. In brief, mesoporous silica nanoparticles (MSN) loaded with doxorubicin (DOX) and phorbol 12-myristate 13-acetate (PMA) were employed as drug carriers and can be specifically opened by nucleolin in a HeLa cell. Then, PMA induced the HeLa cell to generate reactive oxygen species (ROS) and realized ECL imaging of nucleolin. Afterwards, ROS damages DNA and proteins of the tumor cell and doxorubicin induces the apoptosis of HeLa cells by inhibiting the synthesis of genetic material. Finally, DOX and ROS in a synergetic pathway kill HeLa cells efficiently. Kiani et al. [158] reported a bipolar electrode array based on luminol-platinum nanoparticles. These nanoparticles were modified with various monobases and served as ECL probes for detection of mismatches or single nucleotide polymorphisms (SNPs) associated with human diseases. The DNA probe was attached to the anodic poles of the array. When the electrodes were exposed to the monobase-luminol-platinum nanoparticle, hybridization occur. The ECL signal was measured with a digital camera. This biosensor can detect SNPs in the range of 2−600 pM. This simple instrument was applied for the discrimination between Polymerase Chain reaction (PCR) DNA samples of normal, heterozygous and homozygous beta thalassemia genetic disorders. It showed promise as an inexpensive and efficient method for detecting genetic disorders.

As described above, a strategy recently used to improve the ECL response is the introduction of nanomaterials is to increase the sensitivity and therefore extend the applications of ECL sensing.

Nanomaterials present different sizes, morphologies and chemical composition with excellent chemical and optical properties, converting them to good candidates for their use in ECL biosensors. They can be incorporated into the device to improve efficiency, but they also represent a new class of ECL emitters. A novel highly efficient ECL sensor based on reductive Cu(I) particles catalyzed Zn-doped MoS_2_ QDs for human papilloma virus (HPV16 DNA) or cervical cancer detection has been recently developed [159]. In this work, the sulfur vacancies controlled with zinc doping led to the adsorption and coordination with transition metals. Cu(I) particles were prepared to catalyze H_2_O_2_ (co-reactant) in the ECL system. A 4.5-fold enhancement of the signal of Zn-doped MoS_2_ QDs was obtained with the assistance of reductive Cu(I) particles. The biosensor achieved HPV 16 DNA sensitive determination with a limit of detection of 0.03 nmol/L. A smartphone can capture and process the ECL signal by self-developed software into high-resolution imaging, which provides the possibility of developing a point-of-care HPV 16 DNA determination in the future.

Yuan, R. et al. also reported a signal “off–on” microRNA-155 ECL platform based on carboxyl functionalized-poly (9,9-di-n-octylfluorenyl-2,7-diyl) polymer dots (PFO Pdots) [160]. Initially, PFO Pdots were immobilized onto the electrode trough their carboxylic groups to capture DNA duplex track-locker. In the presence of H_2_O_2_, the ECL signal of PFO Pdots was quenched to obtain a signal-off state. Then, the DNA walker, obtained through the target miRNA-155-triggered catalytic hairpin assembly (CHA), walked along the DNA duplex track-locker to output amounts of G-rich short chain, forming a hemin/G-quadruplex. The ECL signal is restored to a signal-on state with the consumption of H_2_O_2_ by hemin/G-quadruplex, achieving an ultrasensitive miRNA-155 detection. The integration of excellent ECL performance of PFO Pdots have provided an ECL emission and an attractive ECL platform for clinical diagnosis with highly efficient quenching effect of H_2_O_2_ without O_2_ as co-reactant or exogenous species.

### 4.2. ECL DNA Biosensors Based on SPEs

As described above, ECL DNA biosensors have a wide range of applications in different areas as medical diagnosis, food analysis and biowarfare agent detection (virus and bacteria), among others. Screen-printing technology for ECL DNA biosensors is one of the most promising approaches towards simple, rapid and inexpensive production of disposable sensing devices intended for use at point of care. In recent years, SPEs with low cost and mass production using thick film technology have been extensively employed for developing novel sensing platforms with improved performance and application in different areas.

Currently, medical diagnosis is a research area of great interest. Reviewing the ECL DNA biosensors trends in this area, it is evident that there is a continued increase of reports in the last years. In this sense, cancer biomarker as miRNA detection is one of the hot topics.

**Figure 10:**
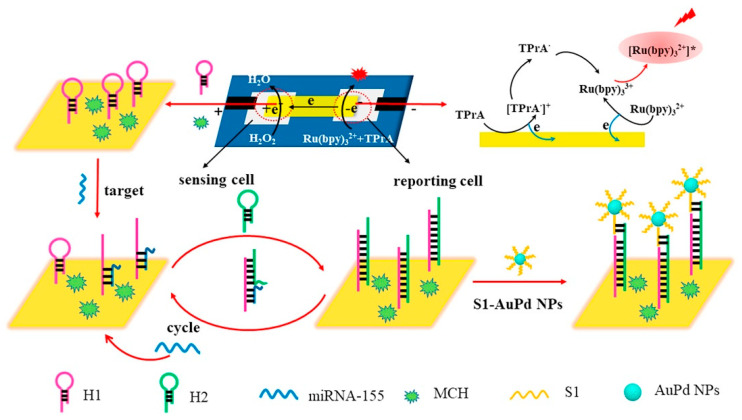
Scheme of ECL biosensor development for the detection of miRNA-155 using a microfluidic system based on a paper-based closed Au-bipolar electrode proposed by Yu et al. [166]. Reproduced and adapted with permission of Elsevier.

MicroRNAs (miRNAs) are a class of endogenous non code small molecules (18–22 nt), which play important roles as significant regulators of fundamental cellular procedures. Recently, abnormal expression of miRNAs has been found in a variety of tumors, which is closely related to the progress, clinical treatment and prognosis of tumors [161,162]. Therefore, miRNAs are becoming as new targets for early cancer diagnosis and treatment [163,164,165]. Currently, simple and sensitive methods for miRNAs detection are still highly demanded. Figure 10 illustrates a novel [Ru(bpy)_3_]^2+^-based ECL immunosensor for rapid and sensitive detection of miRNA-155 utilizing a microfluidic system based on a paper-based closed Au-bipolar electrode (BPE) [166]. Wax-printing technology, screen printing method and in-situ AuNPs growth were used for the microfluidic platform fabrication. DNA (S1)–AuPd nanoparticles was used to modify the cathode of bipolar electrode with a by hybridization chain reaction, in which the target initiated multiple cycles reaction to load high amounts of AuPd nanoparticles that in turn catalyzed H_2_O_2_ reduction. Additionally, the anode of the bipolar electrode is coupled to the [Ru(bpy)_3_]^2+^/TPrA ECL system. Due to the charge balance between the anode and the cathode of BPE, the ECL signal was enhanced. The ECL signal correlated quantitatively with the concentration of miRNA-155 up to 10 μM with a detection limit of 0.67 pM providing a novel way for highly sensitive miRNA-155 detection in clinical application.

**Figure 11:**
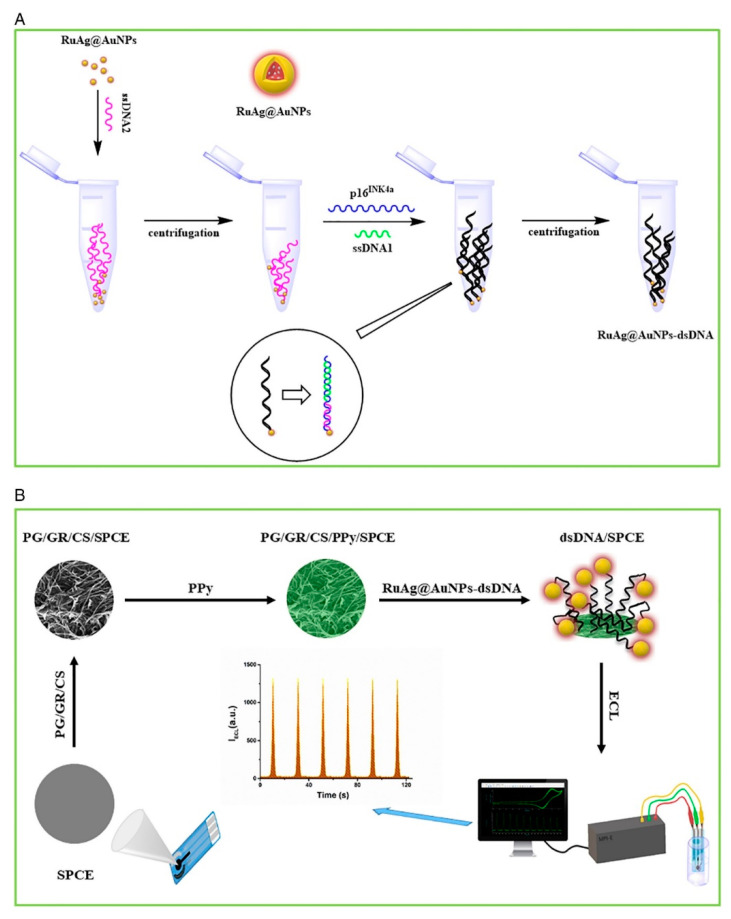
Schematic representation of the DNA-based ECL biosensor for detection of p16INK4a gene using functional paste-like nanofibers composites-modified carbon SPE, developed by Xu et al. [167]. Reproduced and adapted with permission of Elsevier. (**A**) RuAg@AuNPs-dsDNA preparation; (**B**) Paste-like nanofibers composites formation, electropolymerization on the modified carbon SPE surface and dsDNA immobilization.

Another important analyte for medical porpoises is the p16INK4a gene, which is a classical tumor suppressor gene that inhibits the cyclin D-dependent protein kinases. Xu et al. [167] reported on an ECL biosensing system using functional paste-like nanofibers composites-modified carbon SPE for sensitive detection of p16INK4a gene. Figure 11 shows a schematic representation of the ECL DNA biosensor development. Figure 11A shows the dsDNA formation. Firstly, the [Ru(bpy)_3_]^2+^/silver nanoparticles (AgNPs) doped gold core–shell luminescent composite nanoparticles were labeled with ssDNA2 (RuAg@AuNPs-ssDNA2). Afterwards, hybridization reaction of ssDNA1 with the corresponding sequence of p16INK4a gene takes place forming RuAg@AuNPs-dsDNA particles. Figure 11B shows the paste-like nanofibers composites formation using electrospun nanofibers, graphene (GR) and chitosan (CS), which serves as the nanosized backbones for pyrrole (Py) electropolymerization on the modified carbon SPE surface. The composites were used as a substrate for dsDNA immobilization. The ECL intensity is linear to the p16INK4a gene concentration in the range from 0.1 pM to 1 nM, with a detection limit of 0.05 pM.

**Figure 12:**
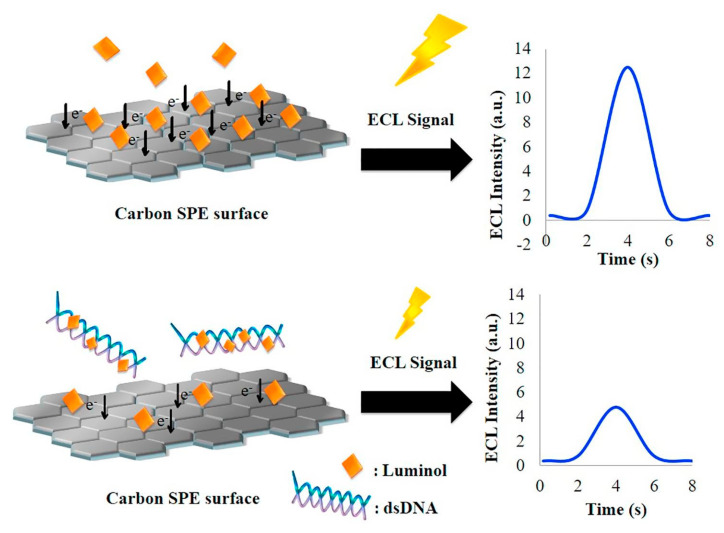
Schematic diagram of DNA ECL immunosensor development for detection of Susscrofa (Porcine) reported by Azam, N.F.N. et al. [169]. Reproduced and adapted with permission of Elsevier.

Nowadays, there is a growing concern of consumers on the authenticity of food ingredients including adulteration. For this reason, the development of low-cost bioanalytical devices that enable high throughput and performance assays in small point-of-analysis will be a priority to improve the quality of food and health. Therefore, researchers have been focusing on developing new detection procedures and tools [168]. In this sense, the combination of an oligonucleotide as recognition element for a specific analyte and of ECL as a readout method has proven to be a valuable strategy for sensitive and specific analytical detection. Figure 12 illustrates the development of a rapid, simple and sensitive luminol-based ECL biosensors for Susscrofa (Porcine) DNA detection [169]. Firstly, porcine DNA was amplified using the loop-mediated isothermal amplification (LAMP) method and then added to luminol solution for the ECL analysis quantification. The DNA-luminol complexes formed slow down the diffusion of luminol towards the electrode surface resulting in low luminol intensity. The LAMP-ECL biosensor shows a sensitive and highly specific detection of Susscrofa (Porcine) DNA detection with a rapid response (around 5 min) and a detection limit of 0.1 pg/μL This approach used in combination with carbon SPE has a great potential to compact biosensors development for food authenticity control.

A new reported application of ECL DNA biosensors based on SPEs is biowarfare agent detection. These agents, which include bacteria, viruses and ortoxins derived from living organisms, are used for bioterrorism purposes and pose an ever-increasing security risk to military and civilian populations [170,171,172]. For this reason, the development of multiplex ECL assays for biowarfare agent is of great interest. O´Sullivan et al. [173] reported the develop of a multiplex detection ECL DNA sensor for the simultaneous detection of six pathogens: *Brucella, melitensis, Bacillus anthracis, Burkholderia mallei, Francisella tularensis, Bacillus thuringiensis var. kurstaki* and *Coxiella burnetiid*. A carbon SPE array was used to develop the assay. This array comprised 42 individual working electrodes with shared counter and reference electrodes. Activated-carbon electrodes were modified covalently with DNA capture probes and then hybridized to target strands. Afterwards, pathogens detection was achieved via sandwich-type format, using [Ru(bpy)_3_]^2+^ labeled reporter probes that were hybridized to the probe–target complexes. An automated microsystem made in a custom designed ECL detection box with integrated electronics, movable photomultiplier detector and fluidics was performed. The detection limits were found to be 0.6–1.2 nmol/L for six targets (from 50 to 122 base pairs) and linear ranges up to 15 nmol/L. The developed system allows the detection in single chip of six targets at sub-nanomolar concentrations.

In conclusion, ECL DNA biosensors based on screen printed technology are successful analytical platforms with high sensitivity and excellent selectivity that are starting to be used for the detection of important analytes, such as viruses, toxins and bacteria (see Table 3). However, the development of new improved devices can be considered as a new research line that is at its beginnings, since most of the reported works have been recently published. There are still several challenges that must be addressed in order to develop devices that have the analytical characteristics required to solve the problems that society demands today.

## 5. Conclusions

ECL biosensors based on screen-printed electrodes are currently commercially successful analytical platforms with high sensitivity and excellent selectivity that are used for the detection of a wide range of analytes, such as biomarkers, toxins, viruses, bacteria and metal ions. The unique and attractive properties of the ECL technique and the popularization of the commercial instrumentation, together with the recent introduction of screen-printed electrodes as electrochemical platforms have paved the way for the development of new designs and applications in clinical prognostics and diagnostics, food and environmental control, biodefense, etc. We overview different applications of ECL biosensing systems in which the electrode employed is a disposable screen-printed electrode. We discuss ECL immunosensors, enzymatic and DNA biosensor. The development of new ECL light-emitting molecules and co-reactants, new ECL mechanisms, disposable electrodes with diverse configurations, miniaturization of instruments and ECL imaging techniques demonstrates that in the future the development of ECL technology will benefit from the rapid and continuous current progress. This in part is due to the advantages that the disposable screen-printed electrodes provide to the system. In particular, recent developments of ECL include fabrication of portable devices for medical point-of-care and field instruments for use in environmental research. Furthermore, the recent incorporation of nanotechnology will improve the efficiency of ECL biosensors based on screen-printed electrodes, since they can act as electrode modifiers giving nanostructured surfaces or as co-reactants in the ECL process.

## Figures and Tables

**Table 1:** Analytical parameters of SPE-based ECL Immunosensors.

Luminophore	Analyte	Sensing Type	Detection Limit	Reference
[Ru(bpy)_3_]^2+^	beta 2-microglobulin	Direct immunosensor-Label-free immunosensor CdSe QDs-Co-reactant AuNPs decorated carbon nano-onions- amplified the ECL signals TPrA-Co-reactant	1 fg/mL	[64]
[Ru(bpy)_3_]^2+^	*Clostridium Perfringens*	Sandwich type immunosensor Pt Nps as luminophore support Ferrite particles support detection antibody and Glucose Dehydrogenase	10^2^ CFU/mL	[66]
[Ru(bpy)_3_]^2+^	human, rabbit and Goat immunoglobulins	Competitive immunoassay Detection antibody covalently bonded to [Ru(bpy)_3_]^2+^	2.9, 6.1 and 6.5 ng/mL	[67]
[Ru(bpy)_3_]^2+^	antitransglutaminase type-2 antibodies	Indirect immunosensor Biotinylated Detection antibody linked to streptavidin-[Ru(bpy)_3_]^2+^	0.47 ng/mL	[68]
[Ru(bpy)_3_]^2+^	carbohydrate antigen 199	Sandwich type immunosensor Detection antibody bonded to AuNPs covered by [Ru(bpy)_3_]^2+^	0.0055 U/mL	[52]
[Ru(bpy)_3_]^2+^	*Francisella tularensis*	Sandwich type immunosensor silica-encapsulated [Ru(bpy)_3_]^2+^ bonded to detection antibody	70 CFU/mL	[69]
[Ru(bpy)_3_]^2+^	prostate specific antigen (PSA), prostate specific membrane antigen (PSMA) and platelet factor-4 (PF-4)	Sandwich type immunosensor Silica nanoparticles coated with [Ru(bpy)_3_]^2+^ bounded to the detection antibody	300–500 fg/mL	[70]
[Ru(bpy)_3_]^2+^	MCF-7 cell	Direct immunosensor Silica and gold nanoparticles bounded to [Ru(bpy)_3_]^2+^	30 Cell/mL	[71]
[Ru(bpy)_3_]^2+^	alpha-fetoprotein	Sandwich type immunosensor Silica and gold nanoparticles bounded to [Ru(bpy)_3_]^2+^Biotinylated capture antibody linked to streptavidin-coated magnetic particles	0.02 ng/mL	[72]
[Ru(bpy)_3_]^2+^	hemoglobin	Molecularly imprinted polymer- antibody Sandwich type immunosensor [Ru(bpy)_3_]^2+^@silica@AuNPs conjugated with the detection antibody magnetic molecularly imprinted polymers	0.023 pg/mL	[73]
[Ru(bpy)_3_]^2+^	carbohydrate antigen-199 and carcinoembryonic antigen	Molecularly imprinted polymer- antibody Sandwich type immunosensor [Ru(bpy)_3_]^2+^@silica@AuNPs conjugated with the detection antibody	0.01 U/L and 0.02 pg/mL	[74]
[Ru(bpy)_3_]^2+^	alpha-fetoprotein	Sandwich type immunosensor [Ru(bpy)_3_]^2+^-MWCNT-AuNPs-Detection antibody Fe3O4@Au nanoparticles linked to capture antibody	3 pg/mL	[75]
[Ru(bpy)_3_]^2+^	mouse IgG	Competitive immunosensor amorphous carbon nanoparticles with quenching effect covalently linked to mouse IgG	0.35 ng/mL	[76]
[Ru(bpy)_3_]^2+^	thyroglobulin	Sandwich type immunosensor TiO_2_ nanodots modified electrode, where the capture antibody is immobilized. Bioconjugate formed by MXenes layers covered by [Ru(bpy)_3_]^2+^ and ZnO quantum dots, linked to the detection antibody	1 fg/mL	[77]
Luminol	human immunodeficiency virus type 1 antibody (HIV-1)	Competitive immunosensor Molecularly imprinted polymer as capture probe Horse radish peroxidase-HIV-1	1:60,000 dilution ratio of standard positive serum	[78]
Luminol	alpha-fetoprotein	Sandwich type immunosensor Luminol groups adsorber over AuPd NPs linked to detection antibody	0.005 ng/mL	[79]
Luminol	carcinoma antigen 125	Sandwich type immunosensor Luminol labeled detection antibody	0.0074 U/mL	[80]
Luminol	transferrin (TRF)	Label-free immunosensor Enhacement of luninol ECL when TRF is attached to the capture antibody	0.033 ng/mL	[82]
Semiconductor graphite-like carbon nitride/S_2_O_8_^2−^ co-reactant	carbohydrate antigen 125	Label-free immunosensor Carboxilate g-C_3_N_4_/aminated Fe_3_O_4_/capture antibody	0.4 mU/mL	[83]
Semiconductor carbon nanocrystals (CNCs)/S_2_O_8_^2−^ co-reactant	prostate specific antigen (PSA)	Sandwich type immunosensor PtAg@CNCs linked to detection antibody	0.6 pg/mL	[84]
CdTe quantum dots/S_2_O_8_^2−^ co-reactant	carcinoembryonic antigen	Sandwich type immunosensor Detection antibody linked to CdTe quantum dots/AgNPs	2.5 mU/mL	[86]
SnO_2_ nanocrystal/S_2_O_8_^2−^ co-reactant	carcinoembryonic antigen	Sandwich type immunosensor SnO_2_@PtRu linked to detection antibody	0.72 pg/mL	[87]
ZnO quantum dots/S_2_O_8_^2−^ co-reactant	prostate specific antigen (PSA)	Sandwich type immunosensor ZnO@CNT linked to detection antibody	0.61 pg/mL	[88]
CdTe quantum dots (QDs)	carcinoma embryonic antigen	Sandwich type immunosensor CdTe QDs linked to detection antibody Capture antibody linked to AuNPs@Fe_3_O_4_ particles.	0.38 pg/mL	[89]
graphene quantum dots (GQDs)/H_2_O_2_ as co-reactant	carcinoma embryonic antigen	Sandwich type immunosensor Au@Pt core–shell nanoparticles modified with graphene quantum dots (GQDs)	0.6 pg/mL	[90]
Carbon dots/TEA as co-reactant	cancer antigen 125	Sandwich type immunosensor Detection antibody linked to carbon nanodots modified nanoporous silica nanoparticles	4.3 mU/mL	[91]
phenyleneethynylene derivatives (P-acid)/TEA as co-reactant	carcinoembryonic antigen	Sandwich type immunosensor P-acid/Pt–AgANPs linked to detection antibody	0.3 pg/mL	[92]

**Table 2:** Analytical parameters of SPE-based ECL enzymatic biosensors.

Luminophore	Enzyme	Analyte	Sensing Type	Detection Limit	Reference
Luminol	Glucose oxidase	Glucose	Surface-unpassivated CdTe QDs	0.3 mM	[108]
Luminol	Glucose oxidase	Glucose	Au and TiO_2_ nano-composite	0.22 mM	[109]
Luminol	Uricase	Uric acid	3,3,5,5-tetramethylbenzidine and chitosan	0.44 µM	[111]
Luminol	Lactate oxidase	Lactate	Methocel membrane	5 µM	[114]
Luminol	Choline oxidase	Choline	Poly(vinyl alcohol) bearing styrylpyridinium groups (PVA–SbQ) photocrosslinked polymer	0.2 µM	[120]
Luminol	Glucose oxidase and Lactate oxidase	Glucose Lactate	PVA–SbQ (poly(vinyl alcohol)	3 µM 10 µM	[121]

**Table 3:** Analytical parameters of SPE-based ECL DNA biosensors.

Luminophore	Analyte	Sensing Type	Detection Limit	Reference
[Ru(phen)_3_]^2+^	mRNA-21	Toehold-mediated strand displacement (TMSD)	0.65 fM	[156]
Luminol	nucleolin in a single HeLa cell	Mesoporous silica nanoparticles (MSN) loaded with doxorubicin (DOX) and phorbol 12-myristate 13-acetate (PMA)		[157]
Luminol	SNP detection	Luminol-platinum nanoparticles	2−600 pM	[158]
Nanomaterials: MoS2 QDs	HPV16 DNA	Cu(I) reductive particles catalyzed Zn-doped MoS2 QDs	0.03 nmol/L	[159]
Nanomaterials: PFO Pdots	microRNA-155	Quenching effect of H_2_O_2_	12.2 aM	[160]
[Ru(phen)_3_]^2+^/TPrA	mRNA-21	Wax-printing technology, screen printing method and in-situ AuNPs growth	0.67 pM	[166]
[Ru(bpy)_3_]^2+^/silver nanoparticles (AgNPs)	p16INK4a	Paste-like nanofibers composites-modified carbon SPE	0.05 pM	[167]
Luminol	Susscrofa (Porcine) DNA	Loop-mediated isothermal amplification	0.1 pg/μL	[169]
[Ru(bpy)_3_]^2+^	pathogens	Sandwich-type assay	0.6–1.2 nmol/L	[173]
